# HER-2/*neu *amplification testing in breast cancer by Multiplex Ligation-dependent Probe Amplification: influence of manual- and laser microdissection

**DOI:** 10.1186/1471-2407-9-4

**Published:** 2009-01-05

**Authors:** Cathy B Moelans, Roel A de Weger, Chantal Ezendam, Paul J van Diest

**Affiliations:** 1 Department of Pathology, University Medical Centre Utrecht, PO Box 85500, Heidelberglaan 100, 3508 GA Utrecht, the Netherlands

## Abstract

**Background:**

Accurate assessment of HER-2/*neu *status is crucial for proper prognostic information and to offer direct appropriate treatment for breast cancer patients. Next to immunohistochemistry (IHC) to evaluate HER2 protein overexpression, a second line gene amplification test is generally deemed necessary for cases with equivocal protein expression. Recently, a new PCR based test, called Multiplex Ligation-dependent Probe Amplification (MLPA), was introduced as a simple and quick method to assess HER-2/*neu *gene amplification status in invasive breast cancer. MLPA was previously shown to correlate well with IHC and *in situ *hybridization (ISH), but a low tumor percentage in the tissue tested could negatively affect the accuracy of MLPA results.

**Methods:**

To examine this, MLPA was repeated in 42 patients after serial H&E section guided manual dissection with a scalpel and after laser microdissection of the tumor.

**Results:**

Both dissection techniques led to higher HER2 gene copy number ratios and thereby made MLPA more quantitative. Concordance between MLPA and ISH improved from 61% to 84% after manual microdissection and to 90% after laser microdissection.

**Conclusion:**

Manual and laser microdissection similarly increase the dynamic range of MLPA copy number ratios which is a technical advantage. As clinically a dichotomization between normal and amplified suffices and MLPA is relatively unsensitive to tumor content, microdissection before MLPA may not be routinely necessary but may be advisable in case of very low tumor content (≤30%), when MLPA results are equivocal, or when extensive ductal carcinoma *in situ *is present. Since differences between manual and laser microdissection were small, less time consuming manual microdissection appears to be sufficient.

## Background

HER-2/*neu *is a proto-oncogene located on chromosome 17q21 encoding a 185 kD transmembrane protein that is involved in signal transduction [1,2]. HER2 belongs to the human epidermal growth factor receptor (EGFR) family and is amplified in about 10–20% of breast carcinomas causing an increased expression of its protein [3-5]. Patients having this overexpression respond well to treatment with trastuzumab, a recombinant humanized monoclonal anti-HER2 antibody [6,7]. Since the costs for trastuzumab therapy are high and side effects are significant, accurate selection of eligible patients for this therapy is very important. Furthermore, amplification of HER2 has also been shown to correlate with poor prognosis [8] and with resistance to conventional adjuvant chemotherapy and tamoxifen [9-13]. With the recognition of its prognostic, predictive and therapeutic implications, assessment of HER2 status has now become of major importance in clinical practice for breast cancer patients.

At present, HER2 status is most commonly assessed by immunohistochemistry (IHC) and/or gene amplification tests such as fluorescence *in situ *hybridization (FISH) [14-16] or chromogenic *in situ *hybridization (CISH) [17]. However, these techniques can only be assessed semi-quantitatively, and amplification detection by easier quantitative PCR techniques has therefore been proposed as an alternative. One of the newly introduced techniques for detection of HER2 amplification is multiplex ligation-dependent probe amplification (MLPA)[18]. In MLPA reactions, mixes composed of up to 45 probes can be used which makes it easy to quantitatively assess the copy number of different genes simultaneously, allowing for multiple target probes and controls. Moreover, this technique requires only minute quantities of short DNA fragments, which makes it very suitable for DNA isolated from paraffin embedded material. In previous studies using whole tissue sections we obtained very promising results with MLPA in comparison with IHC [19], FISH and CISH [20]. However, the dynamic range of MLPA copy number ratios was lower than with FISH. Furthermore, although results showed that amplification could even be detected in cases with a tumor percentage lower than 10%, the sensitivity of MLPA in these cases will depend on the degree of amplification, so lower levels of amplifications can be missed in case of a low tumor percentage. Laser-based tissue microdissection can potentially solve this issue [21]. However, it is relatively time consuming and therefore not very attractive as a routine test, so the question is whether faster H&E guided manual microdissection with a scalpel ("mesodissection") would suffice.

The aim of this study was therefore to determine to which extent manual and laser microdissection improve the dynamic range of copy number ratios and the sensitivity for amplification detection of HER2 by MLPA.

## Methods

### Patient material

Resection specimens were chosen from a previously used series of 423 consecutive invasive breast cancer patients collected between November 2004 and June 2006 at the Department of Pathology of the University Medical Centre in Utrecht. This study using left over material was approved by the Tissue Science Committee of the UMC Utrecht. All tissue samples had already been analyzed by MLPA and IHC and a smaller fraction by *in situ *hybridization (ISH) for HER2 amplification status[20]. From this series, thirty one samples with low tumor content (< 60%) and/or discrepant results between MLPA and IHC/ISH were selected to study whether concordance with ISH (as gold standard) would improve after microdissection. In addition, 11 MLPA-amplified cases were selected to examine whether the dynamic range of HER2 gene copy number ratios increases after microdissection. Tumor percentages were between 10 and 90%. Although MLPA was shown to work well on biopsies in our previous study[20], we selected for this study only resection specimens to be sure to have sufficient material after recutting paraffin blocks.

### Microdissection

Microdissection was performed on 4 μm thick paraffin sections. For manual microdissection, the relevant area was scraped off with a scalpel by comparing with a serial H&E stained slide where tumor tissue was marked and presence of ductal carcinoma *in situ *(DCIS) was noted. For laser microdissection, sections were baked at 56°C for 1 hour, deparaffinized in xylene for 10 minutes and rehydrated through graded alcohols (100%, 85% and 70% for 1 minute each). After staining with haematoxylin for 5 seconds, slides were rinsed in water and dipped in eosin for 5 seconds. Finally, slides were dehydrated in 100% ethanol for 1 minute and air dried. At this point PALM Liquid Cover Glass (LiquidCoverglass, PALM AG, Bernried, Germany) was applied by aerosol to improve morphology and to allow larger tissue areas to be laser pressure-catapulted [22], and sections were air dried for at least 30 minutes. A microdissection system with UV laser (PALM Microlaser Technologies AG, Bernried, Germany) was used to separate between 3 and 40 square mm of invasive tumor groups from their surrounding tissue. Subsequently, these groups were catapulted by laser pressure catapulting into a cap of a common microfuge tube moistened with a drop of mineral oil.

### Multiplex ligation-dependent Probe Amplification (MLPA)

DNA from dissected tumor was isolated by 1 hour incubation in proteinase K (10 mg/ml; Roche, Almere, Netherlands) at 56°C followed by boiling for 10 minutes. This DNA solution (50–100 μl) was, after centrifugation, used in the MLPA analysis according the manufacturers' instructions, using the P004-A1 HER2 kit (MRC Holland, Amsterdam, The Netherlands). This kit contains 3 probes for the HER2 gene, 11 other chromosome 17 control probes, and 25 control probes located on other chromosomes. Details of the probes in this kit can be found at http://www.mrc-holland.com. All tests were performed in duplicate in an ABI 9700 PCR machine. PCR products were analyzed on an ABI310 capillary sequencer (Applied Biosystems, Foster City, CA, USA). HER2 gene copy number was determined by calculating the mean ratio of the HER2 probe peaks with the two previous peaks and the two following peaks. The mean of all three HER2 probe peaks in duplicate (6 values) was calculated. If this value was below 1.5 (cut-off value) the test was scored non-amplified, values 1.5–2.0 were scored as a low level amplification, and values > 2 as HER2 amplified.

### Statistical Analysis

Comparison between copy number ratios before and after microdissection were analyzed by a paired-samples t-test after testing for normal distribution. Association between difference in copy number ratio before and after microdissection and tumor percentage was tested by subtracting ratios and plotting them against tumor percentage, followed by linear regression analysis. All tests were done with SPSS software, regarding two-sided p-values < 0.05 as significant.

## Results

**Figure 1:**
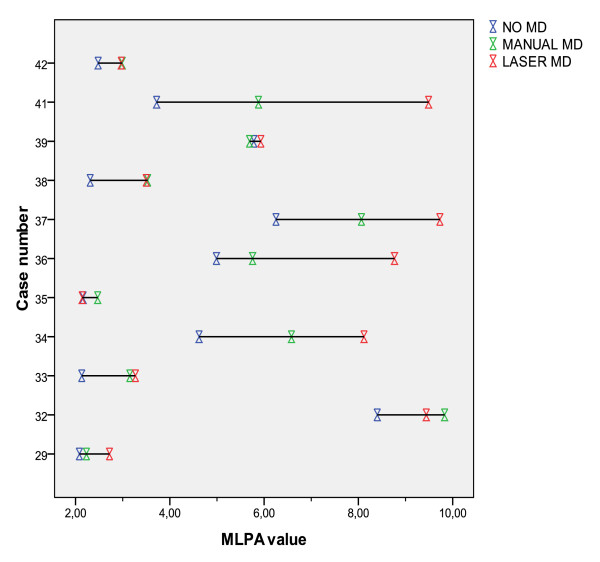
**MLPA values (copy number ratios) for 11 HER2 amplified breast cancer cases before (no MD) and after manual and laser microdissection (MD)**.

**Figure 2:**
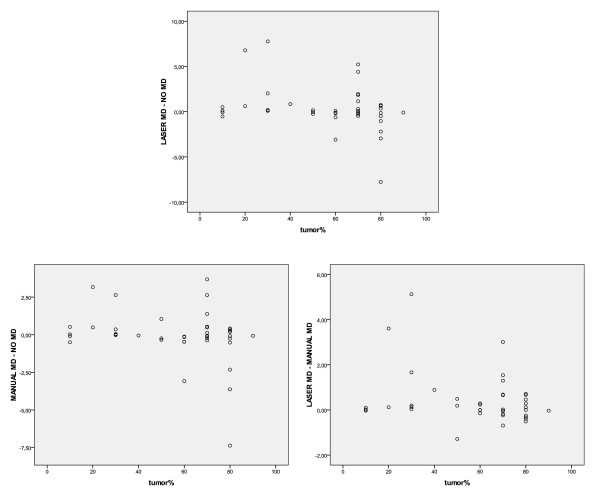
**Scatter plots showing no association between the MLPA ratio difference before (no MD) and after manual- or laser microdissection (MD) on the one hand and tumor percentage of the sample on the other**.

Figure 1 shows the MLPA copy number ratios for 11 MLPA amplified cases before and after microdissection. Manual microdissection led to an increase in measured HER2 gene copy number (p = 0.001), with in most cases a further increase after laser microdissection (p = 0.007 vs non-dissected MLPA), with no significant difference between manual and laser microdissection (p = 0.055). In two cases the presence of DCIS may have caused the laser microdissection value to be lower than the manual microdissection value. Figure 2 shows that there was no association between copy number ratios before and after microdissection and tumor percentage.

Table 1 shows the amplification status of 42 breast cancer patients without and after manual and laser microdissection, in comparison with *in situ *hybridization (ISH) results.

**Table 1:** HER-2/*neu *amplification status by multiplex ligation-dependent probe amplification (MLPA) of 42 breast cancer patients in undissected sections and after manual and laser microdissection, in comparison with *in situ *hybridization and immunohistochemistry.

			MLPA				
Case number	IHC	ISH	Undissected	Manual MD	Laser MD	DCIS	Tumor %
1	0	NA	1	1	1	Yes	30
2	0	NA	1	1	1		30
3	0	NA	1	1	1	Yes	10
4	2	NA	1	1	1		10
5	2	NA	1	1	1	Yes	70
6	2	NA	1	1	2	Yes	70
7	2	NA	1	1	1		50
8	2	NA	1	1	1		70
9	2	NA	1	1	1		70
10	2	NA	1	1	1		60
11	2	NA	1	1	1		30
12	2	NA	1	1	1		60
13	1	LA	2	2	2		70
14	2	A	2	3	3		80
15	2	A	2	2	3	Yes	80
16	3	A	2	2	3		80
17	2	LA	2	2	3		80
18	0	NA	2	1	1	Yes	90
19	0	NA	2	1	1		80
20	1	A	2	3	3		70
21	1	LA	2	3	2	Yes	50
22	1	NA	2	2	1	Yes	80
23	1	NA	2	1	1	Yes	10
24	1	NA	2	1	1	Yes	60
25	0	NA	3	1	1		60
26	0	NA	3	1	1		80
27	1	A	3	3	3		70
28	1	NA	3	3	3	Yes	30
29	3	A	3	3	3		50
30	1	A	3	3	3		80
31	2	A	3	3	3	Yes	70
32	3	A	3	3	3	Yes	80
33	3	A	3	3	3		20
34	3	A	3	3	3		70
35	3	A	3	3	3		80
36	3	A	3	3	3		70
37	3	A	3	3	3		30
38	3	A	3	3	3		70
39	3	A	3	3	3		60
40	2	A	3	3	3	Yes	40
41	3	A	3	3	3	Yes	20
42	3	A	3	3	3		10

IHC = immunohistochemistry (Hercep test), ISH = *in situ *hybridization, NA = non-amplified, LA = low-level amplified, A = amplified, MD = microdissection, DCIS = ductal carcinoma *in situ*

**Figure 3:**
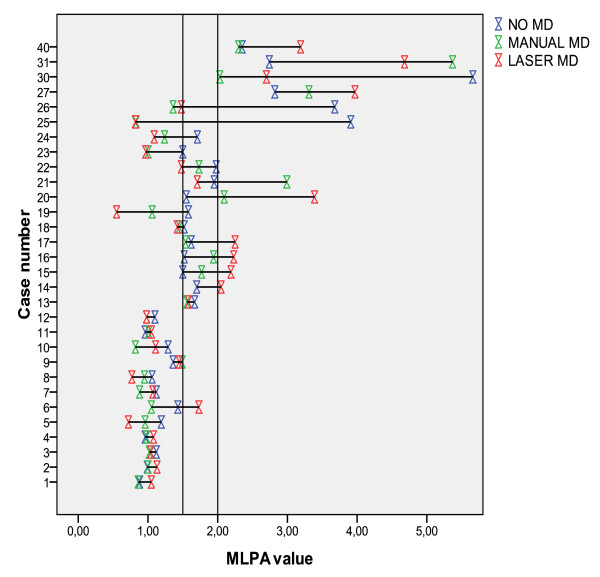
**MLPA values (copy number ratios) for 30 patients before (no MD) and after manual and laser microdissection (MD)**. The vertical lines show the cut-off values (1.50 and 2.00) between an MLPA non-amplified, low-level amplified, and amplified outcome.

Tables 2, 3 and 4 show the concordance between ISH and MLPA without, after manual- and laser microdissection, respectively, with concordance percentages of 61%, 84% and 90%, respectively. For 11/17 patients (65%) that showed discrepancies between MLPA (low or high level) amplified and IHC/ISH, manual or laser microdissection was able to adjust the original MLPA score (based on the whole slide). For 8/11 of these cases (73%), there was no obvious difference between laser microdissection and manual microdissection. However, in 3/11 cases (27%) only laser microdissection was able to change the MLPA outcome. Figure 3 shows that for all but one (11/12) MLPA non-amplified (9 of them IHC equivocal) cases, the MLPA score was unchanged after manual- and laser microdissection. For this case (tumor percentage 70%) the MLPA score became low level amplified after laser microdissection.

**Table 2:** Concordance between HER2 ISH and MLPA without microdissection in 31 invasive breast cancer cases

		MLPA			
		Not amplified	Low level amplified	Amplified	Total
**ISH**	Not amplified	12	5	3	**20**
	Low level amplified	0	3	0	**3**
	Amplified	0	4	4	**8**
	Total	**12**	**12**	**7**	**31**

**Table 3:** Concordance between HER2 ISH and MLPA after manual microdissection in 31 invasive breast cancer cases

		MLPA			
		Not amplified	Low level amplified	Amplified	Total
**ISH**	Not amplified	18	1	1	**20**
	Low level amplified	0	2	1	**3**
	Amplified	0	2	6	**8**
	Total	**18**	**5**	**8**	**31**

**Table 4:** Concordance between HER2 ISH and MLPA after laser microdissection in 31 invasive breast cancer cases

		MLPA			
		Not amplified	Low level amplified	Amplified	Total
**ISH**	Not amplified	18	1	1	**20**
	Low level amplified	0	2	1	**3**
	Amplified	0	0	8	**8**
	Total	**18**	**3**	**10**	**31**

12/31 (39%) samples contained DCIS. In 4/12 (33%) of these cases this could have contributed to biased MLPA results, which was circumvented by manual and/or laser microdissection.

As this paper is in part focussed on discrepant results we also evaluated concordance between the three HER-2/*neu *probes included in the MLPA kit in individual cases, as well as the variation of the control probes between the duplicate assessments. There was full (amplification status) concordance for the three probes in 71% of cases. In the other 29% of cases, the discordant probe ratio value was close but just across the cut-off values. In 62% and 38% of the cases with a discordant probe this concerned a discrepancy between non-amplified/low level amplified and low level/high level amplification, respectively. The third probe accounted for most discordance (48% of discrepancies), possibly due to its lower ratio values compared to the other probes.

Between duplicate measurements, the three probes performed similarly with discrepancies of 9.5%, 11% and 12%, respectively.

As to the 25 reference probes in the P004-A1 MLPA kit (data from the whole original group of unselected patients), amplifications were found in 0.6–10.8% of the patients. High level amplifications of the control probes were very rare, varying between 0 and 2%. Low level amplifications were however common for some probes (range 0.6–9.1%) with 5/25 probes (HIPK3, STCH, CCNB1, PTPN1 and IER3) showing low level amplification in more than 5% of patients.

## Discussion

The goal of our study was to examine the effect of manual and laser microdissection on HER2 MLPA copy number ratios of 42 breast cancer samples with low tumor percentage and/or discrepancies between MLPA on the one hand and IHC and/or ISH on the other. As we wanted to simulate daily practice, we applied a crude method to obtain DNA and did not isolate DNA with more refined methods. We have as yet no indication that a more precise DNA isolation improves HER2 amplification detection by the MLPA technique, but we agree we cannot exclude that. This will be the subject of further research.

Copy number ratios increased after manual microdissection and even more after laser microdissection, indicating that the dynamic range of MLPA increases when the test sample is enriched for tumor cells, making this technique more quantitative. Nevertheless, the highest ratio observed was about 10, which is less than generally observed with FISH. The fact that higher ratios are not observed even after maximal enrichment for tumor cells is probably inherent to the MLPA technique. However, since the copy number ratio, once amplified, does not further contribute to clinical decision making this is not at all a problem in daily practice.

We showed in Figure 2 that there was no association between copy number ratios before and after microdissection and tumor percentage. This can be explained by the presence of background signal from non-tumorous cells (infiltrate/benign breast) and DCIS in non-dissected samples. DCIS can cause a false higher copy number ratio that becomes lower after performing microdissection to exclude the DCIS.

Correlation between MLPA and ISH (as gold standard) improved after manual or laser microdissection, indicating that enrichment for tumor cells increases reliability of MLPA. However, manual or laser microdissection had only an effect on MLPA score in 1/12 non-amplified patients. In this study and in our previous study amplification was detected by MLPA even in cases with a tumor percentage below 10% [20], indicating that MLPA is relatively insensitive to tumor percentage (although sensitivity of MLPA will likely depend on the degree of amplification in case of low tumor percentage) and that routine microdissection may not be required in daily practice. However, also some undissected samples with high tumor % and without DCIS showed amplification by MLPA that could not be confirmed by ISH. Although we cannot exclude that tumor heterogeneity plays a role here, MLPA may occasionally provide false positive results. This concerned however only 6/423 cases, so the rate of false positivity may only be in the range of 1%.

Nevertheless, patients with especially a low level amplification MLPA result seem to benefit most from microdissection. Manual microdissection seems to suffice in most cases as differences between manual and laser microdissection results were small.

The amplification and overexpression of HER2 is seen more frequently in DCIS (50–60%) [23] than in invasive ductal carcinoma of the breast (10–20%) and the presence of DCIS can thereby bias MLPA results. In our study the presence of DCIS probably contributed to biased MLPA outcome as circumvented by manual and/or laser microdissection in 33% (4/12) of (selected) cases. Therefore, if MLPA results are equivocal or extensive DCIS is present, we advise to perform careful manual (or laser) microdissection before MLPA or to use CISH/FISH as an alternative.

When comparing performance of the three HER2 probes, the third probe showed most frequently a discordance with the other two probes, possibly due to its lower ratio values. In duplicate assessments, however, all probes performed about equally well, indicating that reproducibility is good.

Some of the 25 reference probes that were originally chosen for their supposed lack of amplification in breast cancer were nevertheless amplified. Therefore, at least the five probes (HIPK3, STCH, CCNB1, PTPN1 and IER3) that were (low level) amplified in more than 5% of cases may need to be replaced.

## Conclusion

MLPA is a fast, accurate and cheap method to detect breast cancer HER-2/*neu *amplification in small quantities of DNA extracted from paraffin blocks. Amplification can be detected even in cases with very low tumor percentages. Manual or laser microdissection of breast cancer slides before HER2 MLPA may hence not be routinely necessary. However, the dynamic range of the technique improves after manual and laser microdissection and may therefore at least be advisable in case of very low tumour content (≤30%), when the MLPA outcome is equivocal or when extensive DCIS is present. Since differences between manual and laser microdissection were small, less time consuming manual microdissection seems to suffice then.

## Competing interests

The authors declare that they have no competing interests.

## Authors' contributions

CBM and CE carried out the MLPA and microdissection studies. CBM performed the statistical analysis and drafted the manuscript. PJvD and RAdW participated in the design and coordination of the study and helped to draft the manuscript. All authors read and approved the final manuscript.

## Pre-publication history

The pre-publication history for this paper can be accessed here:

http://www.biomedcentral.com/1471-2407/9/4/prepub
